# Factors associated with sexually transmitted infections among care-seeking adults in the African Cohort Study

**DOI:** 10.1186/s12889-021-10762-4

**Published:** 2021-04-16

**Authors:** Michael Semwogerere, Nicole Dear, Joshua Tunnage, Domonique Reed, Hannah Kibuuka, Francis Kiweewa, Michael Iroezindu, Emmanuel Bahemana, Jonah Maswai, John Owuoth, Trevor A. Crowell, Julie A. Ake, Christina S. Polyak, Allahna Esber, for the AFRICOS Study Group, Danielle Bartolanzo, Alexus Reynolds, Katherine Song, Mark Milazzo, Leilani Francisco, Shauna Mankiewicz, Steven Schech, Alexandra Golway, Badryah Omar, Tsedal Mebrahtu, Elizabeth Lee, Kimberly Bohince, Ajay Parikh, Jaclyn Hern, Emma Duff, Kara Lombardi, Michelle Imbach, Leigh Anne Eller, Hannah Kibuuka, Michael Semwogerere, Prossy Naluyima, Godfrey Zziwa, Allan Tindikahwa, Hilda Mutebe, Cate Kafeero, Enos Baghendaghe, William Lwebuge, Freddie Ssentogo, Hellen Birungi, Josephine Tegamanyi, Paul Wangiri, Christine Nabanoba, Phiona Namulondo, Richard Tumusiime, Ezra Musingye, Christina Nanteza, Joseph Wandege, Michael Waiswa, Evelyn Najjuma, Olive Maggaga, Isaac Kato Kenoly, Barbara Mukanza, Jonah Maswai, Rither Langat, Aaron Ngeno, Lucy Korir, Raphael Langat, Francis Opiyo, Alex Kasembeli, Christopher Ochieng, Japhet Towett, Jane Kimetto, Brighton Omondi, Mary Leelgo, Michael Obonyo, Linner Rotich, Enock Tonui, Ella Chelangat, Joan Kapkiai, Salome Wangare, Zeddy Bett Kesi, Janet Ngeno, Edwin Langat, Kennedy Labosso, Joshua Rotich, Leonard Cheruiyot, Enock Changwony, Mike Bii, Ezekiel Chumba, Susan Ontango, Danson Gitonga, Samuel Kiprotich, Bornes Ngtech, Grace Engoke, Irene Metet, Alice Airo, Ignatius Kiptoo, John Owuoth, Valentine Sing’oei, Winne Rehema, Solomon Otieno, Celine Ogari, Elkanah Modi, Oscar Adimo, Charles Okwaro, Christine Lando, Margaret Onyango, Iddah Aoko, Kennedy Obambo, Joseph Meyo, George Suja, Michael Iroezindu, Yakubu Adamu, Nnamdi Azuakola, Mfreke Asuquo, Abdulwasiu Bolaji Tiamiyu, Afoke Kokogho, Samirah Sani Mohammed, Ifeanyi Okoye, Sunday Odeyemi, Aminu Suleiman, Lawrence Umejo, Onome Enas, Miriam Mbachu, Ijeoma Chigbu-Ukaegbu, Wilson Adai, Felicia Anayochukwu Odo, Rabi Abdu, Rosemary Akiga, Helen Nwandu, C Hisara Okolo, Ndubuisis Okeke, Zahra Parker, Asogwa Ugochukwu Linus, Concilia Amaka Agbaim, Tunde Adegbite, Nkenchiere Harrison, Adewale Adelakun, Ekeocha Chioma, Victoria Idi, Rachel Eluwa, Jumoke Nwalozie, Igiri Faith, Blessing Okanigbuan, Achugwo Emmanuel, Nkiru Nnadi, Ndubuisi Rosemary, Uzoegwu Amaka Natalie, Obende Theresa Owanza, Falaju Idowu Francis, Jacintal Elemere, Obilor Ifeoma Lauretta, Edward Akinwale, Inalegwu Ochai, Lucas Maganga, Emmanuel Bahemana, Samoel Khamadi, John Njegite, Connie Lueer, Abisai Kisinda, Jaquiline Mwamwaja, Faraja Mbwayu, Gloria David, Mtasi Mwaipopo, Reginald Gervas, Dorothy Mkondoo, Nancy Somi, Paschal Kiliba, Gwamaka Mwaisanga, Johnisius Msigwa, Hawa Mfumbulwa, Peter Edwin, Willyhelmina Olomi

**Affiliations:** 1 Makerere University Walter Reed Project, Kampala, Uganda; 2 U.S. Military HIV Research Program, Walter Reed Army Institute of Research, Silver Spring, MD USA; 3 Henry M. Jackson Foundation for the Advancement of Military Medicine, Bethesda, MD USA; 4 Strengthening Institutional Capacity for Research Administration (SICRA), Makerere University Lung Institute, Kampala, Uganda; 5 HJF Medical Research International, Abuja, Nigeria; 6 HJF Medical Research International, Mbeya, Tanzania; 7 Kenya Medical Research Institute/U.S. Army Medical Research Directorate – Africa/Kenya, Nairobi, Kenya; 8 HJF Medical Research International, Kericho, Kenya; 9 HJF Medical Research International, Kisumu, Kenya

**Keywords:** Sexually transmitted infections, Sub-Saharan Africa, People living with HIV

## Abstract

**Objectives:**

Sexually transmitted infections (STIs) are a major cause of morbidity. Understanding drivers of transmission can inform effective prevention programs. We describe STI prevalence and identify factors associated with STIs in four African countries.

**Methods:**

The African Cohort Study is an ongoing, prospective cohort in Kenya, Nigeria, Tanzania and Uganda. At enrollment, a physical exam was conducted and STI diagnosis made by a clinician using a syndromic management approach. Multivariable logistic regression was used to estimate adjusted odds ratios (aORs) and 95% confidence intervals (95% CIs) for factors associated with an STI diagnosis.

**Results:**

As of June 2020, 3544 participants were enrolled. STI prevalence was 7.7% and did not differ by HIV status (*p* = 0.30). Prevalence differed by syndrome (3.5% vaginal discharge, 1.5% genital ulcer, 2.1% lower abdominal pain, 0.2% inguinal bubo). The odds of having an STI were higher at all sites compared to Kisumu West, Kenya, and among those with a primary level education or below compared to those with secondary or higher (aOR: 1.77; 95% CI: 1.32–2.38). The odds of an STI diagnosis was higher among participants 18–29 years (aOR: 2.29; 95% CI: 1.35–3.87), females (aOR: 2.64; 95% CI: 1.94–3.59), and those with depression (aOR: 1.78; 95% CI: 1.32–2.38). Among PLWH, similar factors were independently associated with an STI diagnosis. Viral suppression was protective against STIs (aOR: 2.05; 95% CI: 1.32–3.20).

**Conclusions:**

Prevalence of STIs varied by site with young people and females most at risk for STIs. Mental health is a potential target area for intervention.

**Supplementary Information:**

The online version contains supplementary material available at 10.1186/s12889-021-10762-4.

## Key messages

- Syndromically managed STIs are uniquely relevant as clinical diagnosis is the standard of care in most resource-limited settings
- Young people and women were most at risk for STIs in this setting
- Robust mental health programming targeting depression and anxiety may decrease STI transmission in this population

## Introduction

Globally, there is an estimated 376.4 million new cases of curable sexually transmitted infections (STIs), including *Chlamydia trachomatis*, *Neisseria gonorrhoeae*, syphilis and *Trichomonas vaginalis* among people aged 15–49 years, every year [1]. The WHO estimates that sub-Saharan Africa bears approximately 40% of the global burden of STIs [2]. Symptomatic and asymptomatic STIs are a major cause of morbidity in developing countries and can cause infertility, cervical cancer, pelvic inflammatory disease and pregnancy complications [3]. Furthermore, evidence suggests that STIs can increase the risk of acquisition and transmission of HIV [4–7].

Understanding the factors that drive STI transmission is important to implementing effective STI prevention programs, particularly in high HIV-burden settings. Several studies from Sub-Saharan Africa have identified women of reproductive age as at high risk for STIs [8]. Socio-behavioral risk factors for STIs in this group include low levels of education, not being married, multiple sex partners, alcohol and drug use, and early sexual debut [8–11]. Additionally, key populations such as men who have sex with men, transgender women, and commercial sex workers are at increased risk of STI acquisition [12, 13].

Few studies from high HIV-burden settings have addressed STI prevalence and management. The African Cohort Study (AFRICOS) addresses this paucity of data by examining STI diagnoses from four high HIV-burden African countries. In AFRICOS, STIs are diagnosed using a syndromic management clinical approach. In many low- and middle-income countries, including in Africa, a syndromic management approach is often used to diagnose and treat STIs without employing laboratory techniques [14].] Despite substantial drawbacks to this method, including lower sensitivity syndromic management of STIs are uniquely relevant as clinical diagnosis is the standard of care in most resource-limited settings [15, 16].

The aim of this study was to describe STI prevalence and identify factors associated with STI diagnosis in AFRICOS.

## Materials and methods

### Study design and participants

These analyses utilized data from AFRICOS, an ongoing, multi-site, prospective cohort study that has been described previously [17]. In brief, since 2013, AFRICOS has enrolled persons living with HIV (PLWH) and those at risk for HIV in 12 clinical sites across five programs in four countries: South Rift Valley, Kenya; Kisumu West, Kenya; Nigeria; Tanzania; and Uganda. The South Rift Valley site is comprised of six sites- Kericho District Hospital, Tenwek Mission Hospital, Kapkatet District Hospital, AC LITEIN Mission Hospital, Nandi Hills District Hospital, and Kapsabet District Hospital. The Kisumu West, Kenya site is based in the Kisumu West District Hospital, a Ministry of Health District Hospital in Kombewa, Kenya. The Nigerian sites are located in Abuja and Lagos, Nigeria. The Tanzania AFRICOS site is located at the National Institute for Medical Research-Mbeya Medical Research Center. The Uganda site is housed in the Kayunga District Hospital. Participants living with HIV were randomly selected from current client lists or were new enrollees to the clinic with a small subset (9%) recruited from previous HIV research studies. To recruit participants at risk for HIV, adult partners of AFRICOS enrollees living with HIV and seronegative adults from the community or program counseling and testing activities were offered enrollment. All non-pregnant, current clinic patients, 18 years and older, and consenting to data and specimen collection were eligible for inclusion in AFRICOS. This analysis included PLWH and HIV-uninfected participants enrolled in AFRICOS. Patients and the public were not involved in the design or conduct of the study, choice of outcome measures or recruitment to the study.

The study was approved by the institutional review boards of the Walter Reed Army Institute of Research (#1897), Makerere University School of Public Health (#173), Uganda National Committee of Science and Technology (HS-1175), Kenya Medical Research Institute Science and Ethics Review Unit (SSC# 2396, 2371), Tenwek Institutional Ethics Review Committee (SSC# 2371), Tanzania National Institute of Medical Research (NIMR/HQ/R.8a/Vol.1X/1060), Mbeya Medical and Research Ethics Committee (NIMR/HQ/R.8a/Vol.1X/1060), and Ministry of Defense Health Research and Ethics Committee (#3726112019). Research was performed in accordance with the Declaration of Helsinki. All participants gave written informed consent.

### Data collection

At enrollment and biannual follow-up visits, participants received a clinical assessment, and an extensive demographic and socio-behavioral questionnaire was administered by trained study staff. Demographic and socio-behavioral variables included HIV status, age, sex, education, marital status, condom use at last sex, alcohol and drug use, age at first sexual intercourse, number of sexual partners and depression. Depression was assessed at enrollment and subsequent visits using the 20-item Center for Epidemiological Studies-Depression (CES-D) scale [18]. We employed a cutoff point of 16 or greater to identify individuals at risk for clinical depression [19].

Additional variables collected for PLWH included antiretroviral therapy (ART) status, ART adherence based on the self-reported number of days doses were missed in the past month, and HIV-related stigma, defined as experiencing any of the following: social isolation, physical violence, broken family relationships. ART status and regimen history was obtained at enrollment from medical record review and updated prospectively every 6 months. Syphilis testing was performed using country specific guidelines utilizing rapid plasma regain (RPR) as the initial (nontreponemal) screening test. Viral load testing was performed at enrollment and every 6 months for PLWH using nucleic acid amplification methods on one of several testing platforms with lower limit of quantification 20–48 copies/mL [20]. Viral suppression was defined as a viral load < 200 copies/mL.

During the clinical assessment, participants were asked about any symptoms potentially consistent with STIs in the last 2 weeks. An STI was diagnosed by any of the following: vaginal or penile discharge; genital ulcer; blood in urine; burning/painful urination; vaginal itching; painful intercourse; lower abdominal pain; swollen lymph nodes at groin; genital warts; and/or post coital bleeding. We additionally assessed prevalence of the following syndromes: vaginal discharge, genital ulcer, lower back pain, and inguinal bubo [14].

Data were entered and verified in the ClinPlus platform (Anju Software, Tempe, AZ).

### Statistical analyses

Covariates were included based on clinical significance and factors previously identified in the literature to be associated with symptomatic STIs. These included age, sex, HIV status, education level, marital status, age at first sexual intercourse, number of partners, condom use at last sex, alcohol and drug use, and depression. Taking ART or not, ART adherence, stigma, and viral suppression were also considered for the subgroup of PLWH. These analyses were restricted to observations with non-missing data for the outcome variable and all variables included in the fully adjusted models.

Chi-squared tests for categorical variables and student’s t-tests for continuous variables were used to describe differences between those who were and were not diagnosed with an STI at enrollment. Logistic regression analysis was used to estimate unadjusted and adjusted odds ratios (ORs) and 95% confidence intervals (95% CIs) for factors associated with the presence of an STI at the enrollment visit. Backwards stepwise selection with a significance level of 0.10 was used to remove variables from the adjusted model. Modelling was repeated stratifying by study site. To evaluate HIV-specific factors, a subgroup analysis was performed among PLWH.

All analyses were performed in SAS version 9.4 (SAS Institute, Cary, North Carolina) and Stata version 16.0 (StataCorp, College Station, Texas) software.

## Results

### Study population characteristics

From 23 January 2013 to 1 June 2020, 3550 participants had been enrolled into the study, of whom 3544 (99.8%) were complete cases and included in these analyses. Of these 2931 (82.7%) were PLWH and 615 (17.3%) were at risk for HIV. Participants differed in key demographic characteristics by study site including greater proportion of females enrolled in Tanzania (64.0%), younger age in Nigeria, and a higher percentage of participants with at least a secondary education in Nigeria (88.4%) and South Rift Valley, Kenya (50.4%; Supplemental Table 1). STI prevalence did not vary significantly by HIV status but was numerically higher among PLWH as compared to HIV-uninfected participants, 7.9 and 6.7% respectively (*p* = 0.30). Prevalence differed by syndrome (3.5% vaginal discharge, 1.5% genital ulcer, 2.1% lower abdominal pain, 0.2% inguinal bubo).

At enrollment, STI prevalence varied significantly by site, age, education level, sex, marital status, condom use at last sex with regular and casual partners, age at first sexual intercourse, drug use and depression (Table 1). Kisumu, Kenya had the lowest prevalence of STIs with 11 cases (1.8%) while Kayunga, Uganda had the highest prevalence with 136 cases (21.3%; *p* < 0.001). STI prevalence was highest among the youngest participants aged 18–29 years (9.8%) and lowest among the oldest age group, those aged 50 years and older (3.8%; *p* < 0.001). STI prevalence was higher among those with a primary level education or less (8.9% vs 6.1% *p* = 0.002), females (10.4% vs 4.0%; *p* < 0.001), participants who were not married 9.2% vs 6.6%; *p* = 0.004), had no reported drug use (7.9% vs 1.9%; *p* = 0.024) and had depression (11.9% vs 6.7%; *p* < 0.001). STI prevalence was also higher among participants who reported not using condoms with their regular partner (10.9% vs 5.5%; *p* < 0.001), not using condoms with their casual partner (12.7% vs 7.8%; *p* = 0.020) and among those who were 16 years or younger at first sexual intercourse (8.9% vs 7.0%; *p* = 0.042).

**Table 1:** Study Population Characteristics at Enrollment Visit by STI Status

	**All** **(*n*** **= 3544)**	**No STI diagnosis** **(*n*** **= 3271)**	**STI diagnosis** **(*n*** **= 273)**	***p*-value**
**HIV status**				0.30
PLWH	2931 (100.0%)	2699 (92.1%)	232 (7.9%)	
People without HIV	613 (100.0%)	572 (93.3%)	41 (6.7%)	
**Study site**				< 0.001
Kayunga, Uganda	639 (100.0%)	503 (78.7%)	136 (21.3%)	
South Rift Valley, Kenya	1232 (100.0%)	1176 (95.5%)	56 (4.5%)	
Kisumu West, Kenya	631 (100.0%)	620 (98.3%)	11 (1.7%)	
Mbeya, Tanzania	663 (100.0%)	621 (93.7%)	42 (6.3%)	
Abuja & Lagos Nigeria	379 (100.0%)	351 (92.6%)	28 (7.4%)	
**Age at enrollment (years)**				< 0.001
18–29	711 (100.0%)	641 (90.2%)	70 (9.8%)	
30–39	1215 (100.0%)	1109 (91.3%)	106 (8.7%)	
40–49	1063 (100.0%)	987 (92.9%)	76 (7.1%)	
50+	555 (100.0%)	534 (96.2%)	21 (3.8%)	
**Education**				0.002
Primary or below	2004 (100.0%)	1825 (91.1%)	179 (8.9%)	
Secondary or above	1540 (100.0%)	1446 (93.9%)	94 (6.1%)	
**Sex**				< 0.001
Male	1484 (100.0%)	1425 (96.0%)	59 (4.0%)	
Female	2060 (100.0%)	1846 (89.6%)	214 (10.4%)	
**Marital status**				0.004
Not married	1451 (100.0%)	1317 (90.8%)	134 (9.2%)	
Married	2093 (100.0%)	1954 (93.4%)	139 (6.6%)	
**Used condom at last sex with regular** partner				< 0.001
No regular partner	981 (100.0%)	898 (91.5%)	83 (8.5%)	
Used a condom	1590 (100.0%)	1503 (94.5%)	87 (5.5%)	
Did not use a condom	916 (100.0%)	816 (89.1%)	100 (10.9%)	
Missing	57 (100.0%)	54 (94.7%)	3 (5.3%)	
**Used condom at last sex with casual partner**				0.020
No casual partner	3092 (100.0%)	2850 (92.2%)	242 (7.8%)	
Used a condom	279 (100.0%)	266 (95.3%)	13 (4.7%)	
Did not use a condom	118 (100.0%)	103 (87.3%)	15 (12.7%)	
Missing	55 (100.0%)	52 (94.5%)	3 (5.5%)	
**Current number of sexual partners**				0.24
No sexual partners	863 (100.0%)	786 (91.1%)	77 (8.9%)	
One sexual partner	2138 (100.0%)	1985 (92.8%)	153 (7.2%)	
Two or more sexual partners	485 (100.0%)	445 (91.8%)	40 (8.2%)	
Missing	58 (100.0%)	55 (94.8%)	3 (5.2%)	
**Early sexual debut**				0.042
> 16 years at first sex	1998 (100.0%)	1858 (93.0%)	140 (7.0%)	
≤ 16 years at first sex	1440 (100.0%)	1312 (91.1%)	128 (8.9%)	
Missing	106 (100.0%)	101 (95.3%)	5 (4.7%)	
**Consume alcohol**				0.77
No	2827 (100.0%)	2611 (92.4%)	216 (7.6%)	
Yes	716 (100.0%)	659 (92.0%)	57 (8.0%)	
Missing	1 (100.0%)	1 (100.0%)	0 (0.0%)	
**Recreational drug use**				0.024
No	3438 (100.0%)	3167 (92.1%)	271 (7.9%)	
Yes	105 (100.0%)	103 (98.1%)	2 (1.9%)	
Missing	1 (100.0%)	1 (100.0%)	0 (0.0%)	
**Depression** ^a^				< 0.001
No	2872 (100.0%)	2679 (93.3%)	193 (6.7%)	
Yes	672 (100.0%)	592 (88.1%)	80 (11.9%)	
**HIV-related characteristics (among PLWH only)**				
	**All** **(*n*** **= 2915)**	**No STI diagnosis** **(*n*** **= 2683)**	**STI diagnosis** **(*n*** **= 232)**	***p*-value**
**Missed days of ART doses in past month**				< 0.001
On ART, no days missed	1736 (100.0%)	1655 (95.3%)	81 (4.7%)	
On ART, one or more days missed	280 (100.0%)	261 (93.2%)	19 (6.8%)	
Not on ART	899 (100.0%)	767 (85.3%)	132 (14.7%)	
**Stigma** ^b^				0.24
No	2581 (100.0%)	2381 (92.3%)	200 (7.7%)	
Yes	334 (100.0%)	302 (90.4%)	32 (9.6%)	
**Viral suppression**				< 0.001
On ART, vl < 200 copies/mL	1551 (100.0%)	1487 (95.9%)	64 (4.1%)	
On ART, vl ≥ 200 copies/mL	465 (100.0%)	429 (92.3%)	36 (7.7%)	
Not on ART	899 (100.0%)	767 (85.3%)	132 (14.7%)	

All data are presented as n (row percentage). *P*-values were calculated using Chi-squared tests

^a^ Depression was assessed using the 20-item Center for Epidemiological Studies-Depression (CES-D) scale; a cutoff point of 16 or greater was used to identify individuals at risk for clinical depression

^b^ Participants were defined as experiencing stigma if they had experienced any of the following: social isolation, physical violence, broken family relationships

Among the subgroup of PLWH, STI prevalence was higher among those with poor adherence or who were not on ART (6.8 and 14.7% vs 4.7%; *p* < 0.001) and among those who were virally unsuppressed at a level of 200 copies/mL or less (7.7% vs 4.1%; *p* < 0.001).

### Factors associated with STI diagnosis

Factors independently associated with an STI diagnosis at enrollment among all participants included site, age, sex, education level and depression (Table 2). As compared to Kisumu West, Kenya, the odds of being diagnosed with an STI at enrollment were higher for participants at the South Rift Valley Kenyan, Uganda, Tanzanian and Nigerian sites (South Rift Valley, Kenya aOR:3.03; 95% CI 1.56–5.88, Uganda aOR: 16.26; 95% CI 8.65–30.55, Tanzania aOR 3.70; 95% CI: 1.88–7.29, Nigeria aOR: 5.84; 95% CI 2.78–12.28). Participants in the youngest age group, 18–29 years, had higher odds of being diagnosed with an STI at enrollment compared to the oldest age group, those 50 years and older (aOR: 2.29; 95% CI: 1.35–3.87). Females had higher odds of being diagnosed with an STI compared to males (aOR: 2.64; 95% CI: 1.94–3.59). Those with a primary level education or lower had a higher odds of being diagnosed with an STI as compared to those with a secondary level education or higher (aOR: 1.41; 95% CI: 1.05–1.90). Those with depression were had higher odds of being diagnosed with an STI at enrollment (aOR: 1.78; 95% CI: 1.32–2.38).

**Table 2:** Logistic Regression model examining factors associated with prevalent STI at enrollment visit among all AFRICOS participants

	Unadjusted OR	95% CI	Adjusted OR	95% CI
**HIV status**				
HIV-uninfected	Ref			
HIV-uninfected	1.20	0.85–1.69		
**Study site**				
Kayunga, Uganda	**15.24**	**8.15–28.49**	**16.26**	**8.65–30.55**
South Rift Valley, Kenya	**2.68**	**1.40–5.16**	**3.03**	**1.56–5.88**
Kisumu West, Kenya	**–**		**–**	
Mbeya, Tanzania	**3.81**	**1.94–7.47**	**3.70**	**1.88–7.29**
Abuja & Lagos Nigeria	**4.50**	**2.21–9.14**	**5.84**	**2.78–12.28**
**Age at enrollment (years)**				
18–29	**2.78**	**1.68–4.58**	**2.29**	**1.35–3.87**
30–39	**2.43**	**1.50–3.93**	**2.06**	**1.25–3.39**
40–49	**1.96**	**1.19–3.21**	**1.76**	**1.05–2.93**
50+	Ref		–	
**Sex**				
Male	Ref		–	
Female	**2.80**	**2.08–3.77**	**2.64**	**1.94–3.59**
**Education**				
Primary or below	**1.51**	**1.16–1.95**	**1.41**	**1.05–1.90**
Secondary or above	**Ref**		**–**	
**Used condom at last sex with regular partner**				
No regular partner	1.60	1.17–2.18		
Used a condom				
Did not use a condom	1.33	0.98–1.80		
**Used condom at last sex with casual partner**				
No casual partner	1.74	0.98–3.08		
Used a condom	Ref			
Did not use a condom	2.98	1.37–6.48		
**Current number of sexual partners**				
No sexual partners	1.27	0.95–1.69		
One sexual partner	Ref			
Two or more sexual partners	1.17	0.81–1.68		
**Early sexual debut**				
> 16 years at first sex	Ref			
≤ 16 years at first sex	**1.30**	**1.01–1.66**		
**Consume alcohol**				
No	Ref			
Yes	1.05	0.77–1.42		
**Recreational drug use**				
No	Ref			
Yes	**0.23**	**0.06–0.93**		
**Depression**				
No	Ref		–	
Yes	**1.88**	**1.42–2.47**	**1.78**	**1.32–2.38**

Bold indicates significance at *p* < 0.05

Stratifying by site, we found that sex was the only factor included in all models across all sites (Table 3). There were no significant factors associated with STI prevalence in Kisumu West, Kenya due to the limited power with the small number of participants with an STI diagnosis from this site. Participants from the Tanzania and Nigeria sites who had an early sexual debut ≤16 years of age had increased odds of STI at enrollment (aOR Tanzania: 2.91, 95% CI: 1.48–5.70; aOR Nigeria: 3.29, 95% CI: 1.33–8.16) compared to those with a later sexual debut.

**Table 3:** Logistic Regression models examining factors associated with prevalent STI at enrollment visit among all AFRICOS participants and stratified by study site

	Uganda		South Rift Valley, Kenya		Kisumu West, Kenya		Tanzania		Nigeria	
	Adjusted OR	95% CI	Adjusted OR	95% CI	Adjusted OR	95% CI	Adjusted OR	95% CI	Adjusted OR	95% CI
**Age at enrollment (years)**										
18–29			1.96	0.91–4.20					**5.79**	**2.30–14.57**
30–39			1.78	0.95–3.31					–	
40–49			–						–	
50+			Ref						Ref	
**Sex**										
Male	Ref		Ref		Ref		Ref		Ref	
Female	**1.79**	**1.18–2.72**	**4.73**	**2.11–10.61**	6.84	0.87–53.90	**4.28**	**1.49–12.31**	**2.89**	**1.33–8.16**
**Education**										
Primary or below									**7.47**	**2.61–21.32**
Secondary or above									Ref	
**Early sexual debut**										
> 16 years at first sex							Ref		Ref	
≤ 16 years at first sex							**2.91**	**1.48–5.70**	**3.29**	**1.33–8.16**
**Depression**										
No	Ref									
Yes	**2.08**	**1.34–3.22**								

Bold indicates significance at *p* < 0.05

Similar factors were found to be independently associated with prevalent STIs among PLWH, including site, age, and sex (Table 4). Viral suppression at ≥200 copies/mL was found to be protective against STIs as compared to those with a viral load < 200 copies/mL (aOR: 2.06; 95% CI: 1.32–3.21). Additionally, PLWH with depression had a 63% higher odds of being diagnosed with an STI at enrollment than those without depression (aOR: 1.63; 95% CI: 1.18–2.26).

**Table 4:** Logistic Regression model examining factors associated with prevalent STI at enrollment visit among PLWH

	Unadjusted OR	95% CI	Adjusted OR	95% CI
**Study site**				
Kayunga, Uganda	**12.91**	**1.76–7.63**	**10.64**	**5.56–20.34**
South Rift Valley, Kenya	**2.15**	**1.11–4.18**	**2.17**	**1.11–4.25**
Kisumu West, Kenya	**Ref**		**Ref**	
Mbeya, Tanzania	**2.78**	**1.39–5.57**	**2.32**	**1.15–4.68**
Abuja & Lagos Nigeria	**3.67**	**1.76–7.63**	**3.18**	**1.51–6.70**
**Age at enrollment (years)**				
18–29	**3.20**	**1.86–5.51**	**1.83**	**1.02–3.26**
30–39	**2.65**	**1.58–4.45**	**1.88**	**1.09–3.24**
40–49	**1.77**	**1.03–3.03**	1.51	0.86–2.65
50+	Ref		–	
**Sex**				
Male	Ref		–	
Female	**3.02**	**2.17–4.20**	**2.84**	**2.01–4.00**
**Education**				
Primary or below	**1.33**	**1.01–1.76**		
Secondary or above	**Ref**			
**Used condom at last sex with regular partner**				
No regular partner	**1.83**	**1.31–2.55**		
Used a condom	**Ref**			
Did not use a condom	**2.52**	**1.80–3.53**		
**Used condom at last sex with casual partner**				
No casual partner	1.87	0.98–3.59		
Used a condom	Ref			
Did not use a condom	**2.67**	**1.09–6.51**		
**Current number of sexual partners**				
No sexual partners	1.34	0.99–1.80		
One sexual partner	Ref			
Two or more sexual partners	1.02	0.67–1.55		
**Early sexual debut**				
> 16 years at first sex	Ref			
≤ 16 years at first sex	1.24	0.94–1.62		
**Consume alcohol**				
No	Ref			
Yes	1.20	0.87–1.66		
**Recreational drug use**				
No	Ref			
Yes	0.14	0.02–1.00		
**Depression**				
No	Ref		–	
Yes	**1.90**	**1.41–2.56**	**1.63**	**1.18–2.26**
**Missed days of ART doses in past month**				
No days missed	Ref			
One or more days missed	1.49	0.89–2.49		
Not on ART	**3.52**	**2.63–4.70**		
**Stigma**				
No	Ref			
Yes	1.26	0.85–1.87		
**Viral suppression**				
On ART, vl < 200 copies/mL	Ref		**–**	
On ART, vl ≥ 200 copies/mL	**1.95**	**1.28–2.97**	**2.06**	**1.32–3.21**
Not on ART	4.00	**2.93–5.46**	**2.20**	**1.56–3.09**

Bold indicates significance at *p* < 0.05

## Discussion

The overall prevalence of syndromic STIs in this multi-country cohort in four sub-Saharan African countries was 7.7% and did not differ significantly by HIV status. Global estimates based on a systematic review of 130 studies, estimate that in 2016, the prevalence of diagnosed STIs was lower than our findings for symptomatic STIs. However, when restricting to the African region only, prevalence was similar although varied by pathogen [1]. A cross-sectional study in another high-HIV prevalence setting found a higher prevalence of STIs and similar findings of no association with HIV status [8]. Another meta-analysis of 18 HIV prevention studies of women found that the prevalence of these four STIs varied by age and clinic or community setting [21]. Few recent studies have assessed the overall burden of syndromically managed STIs in Africa.

This study found a higher prevalence of symptomatic STIs among female participants compared to males. Other studies conducted in sub-Saharan Africa have similarly found that females have a higher prevalence of STIs compared to males in both surveillance and cross-sectional studies [22, 23]. Females may be at increased risk for STIs for several reasons. Gender inequities may reduce educational and economic opportunities for women and encourage risky sexual behavior such as engagement in transactional sex and decreased bargaining power for condom use during sex [24]. Biological factors such as vaginal microbiota and thinner vaginal lining may also put women at increased risk of STI acquisition compared to men [25]. Women are particularly vulnerable to STI acquisition and should be a priority target population, especially those of childbearing age for whom STIs can cause severe morbidity and pregnancy complications.

The prevalence of STIs was highest among those aged 18–29 years old. Other studies have found elevated STI prevalence among young women across various regions and populations [21]. A study among men who have sex with men and transgender women in Nigeria found that Chlamydia and gonorrhea were about twice as common in participants younger than 30 years old compared to those 30 years and older [26]. It is evident that young people are at high risk for STIs and should be a primary target population for intervention. Other studies have shown that cash transfer programs, whereby individuals are given a sum of money either unconditionally or conditionally on an action like attending school, can be effective in reducing STI infections among young women and should be explored in this population [27, 28].

The interaction of mental health and high-risk sexual behavior is well established in resource rich countries. However, this interaction has been poorly studied in Africa. A prior study of key populations affected by HIV in Nigeria suggested that stigmatizing experiences and mental health issues increase STI risk through pathways that include decreased condom use [29]. We have previously reported that depression was associated with decreased ART adherence and elevated viral loads among AFRICOS participants living with HIV [30]. Our data suggest an association between depression, viral non-suppression and an increased risk of STIs. This potentially represents a robust target for intervention as only 10% of persons living in Sub-Saharan Africa have adequate access to mental health care [31]. Integrating care, such as mental health services and behavioral counseling for STI prevention, may be an effective and cost-effective intervention to reduce STIs [32].

Differences in the burden of STIs observed across clinical care site may reflect other social, structural, or cultural nuances that may affect STI transmission and acquisition but that we were unable to capture adequately. Future studies should interrogate site-specific factors to identify those that could potentially reduce the risk of STIs.

### Limitations

This study helps fill a gap in the literature by examining syndromic management of STIs from four high HIV-burden African countries; however, several limitations of these analyses are acknowledged. The syndromic management approach to STI diagnosis used in this study is uniquely relevant as it is the standard of care in most resource-limited settings. However, it likely underestimates the true STI burden [3, 26]. In addition, participants enrolled in a clinic-based cohort study are more likely to be connected to a healthcare system and may have better access to condoms and counseling on condom use than the general population [33]. We also addressed missing data by using complete cases only which potentially can introduce bias. However, as the data were missing at random we believe that the bias is minimal. Our list of genitourinary symptoms was broader than that utilized in STI syndromic management and thus may be at greater risk of false positives. Lastly, these cross-sectional analyses only captured STI diagnoses at a single point in time. Further analyses should utilize longitudinal data to understand factors associated with incident STIs and persistent infection.

## Conclusion

This study identified individuals at high risk for STIs as well as target areas for potential future intervention. Young people and women were most at risk for STIs and should be a focus of future interventions due to the implications of untreated STIs in pregnant women. Additionally, programs focused on women’s health may improve outcomes in all groups. Persons with depression were also identified to be at higher risk for STIs, thus robust mental health programming targeting depression/anxiety may decrease STI transmission.

## Supplementary Information

**Additional file 1: Supplemental Table 1.** Study Population Characteristics at Enrollment Visit by Study Site.

## Data Availability

The datasets generated and/or analyzed during the current study are not publicly available due to privacy protections but are available from the corresponding author on reasonable request. The Henry M. Jackson Foundation for the Advancement of Military Medicine (HJF) and the Water Reed Army Institute of Research (WRAIR) are committed to safeguarding the privacy of research participants. Distribution of data will require compliance with all applicable regulatory and ethical processes, including establishment and approval of an appropriate data-sharing agreement. To request a minimal data set, please contact the data coordinating and analysis center (DCAC) at PubRequest@hivresearch.org and indicate the RV329 study along with the name of the manuscript.

## Abbreviations

AFRICOS: African Cohort Study
ART: Antiretroviral therapy
CESD: Center for Epidemiological Studies-Depression
CIs: Confidence intervals
OR: Odds ratio
PLWH: People living with HIV
STI: Sexually transmitted infection
