# Supplementary material for: Factors associated with sexually transmitted infections among care-seeking adults in the African Cohort Study
Source: BMC Public Health. 2021 Apr 16;21:738. doi: 10.1186/s12889-021-10762-4 (PMC8052711; doi:10.1186/s12889-021-10762-4)
Supplement: Supplementary file 1 — Additional file 1: Supplemental Table 1. Study Population Characteristics at Enrollment Visit by Study Site. [file 12889_2021_10762_MOESM1_ESM.docx]

Supplemental Table 1: Study Population Characteristics at Enrollment Visit by Study Site

|  | **Kayunga, Uganda** | **South Rift Valley, Kenya** | **Kisumu West, Kenya** | **Mbeya, Tanzania** | **Abuja & Lagos Nigeria** | **p-value** |
| --- | --- | --- | --- | --- | --- | --- |
|  | N=639 | N=1232 | N=631 | N=663 | N=379 |  |
| **HIV Status** |  |  |  |  |  | 0.16 |
| HIV-infected | 526 (82.3%) | 1024 (83.1%) | 506 (80.2%) | 566 (85.4%) | 309 (81.5%) |  |
| HIV-uninfected | 113 (17.7%) | 208 (16.9%) | 125 (19.8%) | 97 (14.6%) | 70 (18.5%) |  |
| **Age** |  |  |  |  |  | <0.001 |
| 18-29 | 156 (24.4%) | 182 (14.8%) | 149 (23.6%) | 157 (23.7%) | 67 (17.7%) |  |
| 30-39 | 206 (32.2%) | 435 (35.3%) | 214 (33.9%) | 197 (29.7%) | 163 (43.0%) |  |
| 40-49 | 187 (29.3%) | 415 (33.7%) | 148 (23.5%) | 196 (29.6%) | 117 (30.9%) |  |
| 50+ | 90 (14.1%) | 200 (16.2%) | 120 (19.0%) | 113 (17.0%) | 32 (8.4%) |  |
| **Sex** |  |  |  |  |  | 0.008 |
| Male | 267 (41.8%) | 523 (42.5%) | 282 (44.7%) | 239 (36.0%) | 173 (45.6%) |  |
| Female | 372 (58.2%) | 709 (57.5%) | 349 (55.3%) | 424 (64.0%) | 206 (54.4%) |  |
| **Education** |  |  |  |  |  | <0.001 |
| Primary or less | 444 (69.5%) | 611 (49.6%) | 455 (72.1%) | 450 (67.9%) | 44 (11.6%) |  |
| Secondary or above | 195 (30.5%) | 621 (50.4%) | 176 (27.9%) | 213 (32.1%) | 335 (88.4%) |  |
| **Used condom at last sex with regular partner** |  |  |  |  |  | <0.001 |
| No regular partner | 202 (31.6%) | 323 (26.2%) | 154 (24.4%) | 216 (32.6%) | 87 (23.0%) |  |
| Used a condom | 159 (24.9%) | 726 (58.9%) | 359 (56.8%) | 212 (32.0%) | 134 (35.4%) |  |
| Did not use a condom | 268 (41.9%) | 171 (13.9%) | 114 (18.0%) | 209 (31.5%) | 154 (40.6%) |  |
| Missing | 10 (1.6%) | 12 (1.0%) | 5 (0.8%) | 26 (3.9%) | 4 (1.1%) |  |
| **Used condom at last sex with casual partner** |  |  |  |  |  | <0.001 |
| No casual partner | 559 (87.5%) | 1130 (91.7%) | 528 (83.5%) | 553 (83.4%) | 323 (85.2%) |  |
| Used a condom | 39 (6.1%) | 64 (5.2%) | 83 (13.1%) | 50 (7.5%) | 43 (11.3%) |  |
| Did not use a condom | 31 (4.9%) | 26 (2.1%) | 18 (2.8%) | 34 (5.1%) | 9 (2.4%) |  |
| Missing | 10 (1.6%) | 12 (1.0%) | 3 (0.5%) | 26 (3.9%) | 4 (1.1%) |  |
| **Current number of sexual partners** |  |  |  |  |  | <0.001 |
| No sexual partners | 177 (27.7%) | 305 (24.8%) | 100 (15.8%) | 209 (31.5%) | 73 (19.3%) |  |
| One sexual partner | 354 (55.4%) | 771 (62.6%) | 445 (70.4%) | 331 (49.9%) | 237 (62.5%) |  |
| Two or more sexual partners | 98 (15.3%) | 144 (11.7%) | 82 (13.0%) | 96 (14.5%) | 65 (17.2%) |  |
| Missing | 10 (1.6%) | 12 (1.0%) | 5 (0.8%) | 27 (4.1%) | 4 (1.1%) |  |
| **Early sexual debut** |  |  |  |  |  | <0.001 |
| >16 years at first sex | 348 (54.5%) | 633 (51.4%) | 269 (42.6%) | 438 (66.1%) | 311 (82.1%) |  |
| <16 years at first sex | 275 (43.0%) | 577 (46.8%) | 352 (55.7%) | 183 (27.6%) | 53 (14.0%) |  |
| Missing | 16 (2.5%) | 22 (1.8%) | 11 (1.7%) | 42 (6.3%) | 15 (4.0%) |  |
| **Consume alcohol** |  |  |  |  |  | <0.001 |
| Yes | 493 (77.2%) | 1083 (87.9%) | 539 (85.3%) | 458 (69.1%) | 255 (67.3%) |  |
| No | 146 (22.8%) | 149 (12.1%) | 93 (14.7%) | 204 (30.8%) | 124 (32.7%) |  |
| Missing | 0 (0.0%) | 0 (0.0%) | 0 (0.0%) | 1 (0.2%) | 0 (0.0%) |  |
| **Recreational drug use** |  |  |  |  |  | <0.001 |
| No | 634 (99.2%) | 1211 (98.3%) | 584 (92.4%) | 645 (97.3%) | 365 (96.3%) |  |
| Yes | 5 (0.8%) | 21 (1.7%) | 48 (7.6%) | 17 (2.6%) | 14 (3.7%) |  |
| Missing | 0 (0.0%) | 0 (0.0%) | 0 (0.0%) | 1 (0.2%) | 0 (0.0%) |  |
| **Depression ^a^** |  |  |  |  |  | <0.001 |
| No | 511 (80.0%) | 1045 (84.8%) | 477 (75.5%) | 522 (78.7%) | 318 (83.9%) |  |
| Yes | 128 (20.0%) | 187 (15.2%) | 155 (24.5%) | 141 (21.3%) | 61 (16.1%) |  |
| **HIV-related characteristics (among PLWH only)** | | | | | |  |
|  | **Kayunga, Uganda** | **South Rift Valley, Kenya** | **Kisumu West, Kenya** | **Mbeya, Tanzania** | **Abuja & Lagos Nigeria** |  |
|  | N=526 | N=1024 | N=506 | N=566 | N=309 |  |
| **Missed days of ART doses in past month** |  |  |  |  |  |  |
| On ART, no days missed | 172 (32.7%) | 733 (71.6%) | 345 (68.2%) | 354 (62.5%) | 143 (46.3%) |  |
| On ART, one or more days missed | 47 (8.9%) | 75 (7.3%) | 56 (11.1%) | 43 (7.6%) | 60 (19.4%) |  |
| Not on ART | 307 (58.4%) | 216 (21.1%) | 105 (20.8%) | 169 (29.9%) | 102 (33.0%) |  |
| **Stigma ^b^** |  |  |  |  |  |  |
| No | 445 (84.6%) | 904 (88.3%) | 448 (88.5%) | 521 (92.0%) | 276 (89.3%) |  |
| Yes | 81 (15.4%) | 120 (11.7%) | 58 (11.5%) | 45 (8.0%) | 33 (10.7%) |  |
| **Viral suppression** |  |  |  |  |  |  |
| On ART, vl<200 copies/mL | 186 (35.4%) | 662 (64.6%) | 336 (66.4%) | 230 (40.6%) | 137 (44.3%) |  |
| On ART, vl>200 copies/mL | 33 (6.3%) | 144 (14.1%) | 61 (12.1%) | 163 (28.8%) | 64 (20.7%) |  |
| Not on ART | 307 (58.4%) | 216 (21.1%) | 105 (20.8%) | 169 (29.9%) | 102 (33.0%) |  |

P-values were calculated using Chi-squared tests.

^a^ Depression was assessed using the 20-item Center for Epidemiological Studies-Depression (CES-D) scale; a cutoff point of 16 or greater was used to identify individuals at risk for clinical depression.

^b^ Participants were defined as experiencing stigma if they had experienced any of the following: social isolation, physical violence, broken family relationships.
